# Development of Next-Generation Nutritionally Fortified Plant-Based Milk Substitutes: Structural Design Principles

**DOI:** 10.3390/foods9040421

**Published:** 2020-04-03

**Authors:** David Julian McClements

**Affiliations:** 1 Department of Food Science, University of Massachusetts Amherst, Amherst, MA 01003, USA; mcclements@foodsci.umass.edu; 2 Department of Food Science & Bioengineering, Zhejiang Gongshang University, 18 Xuezheng Street, Hangzhou 310018, China

**Keywords:** plant-based milks, emulsions, oil bodies, proteins, sustainability

## Abstract

Consumers are increasingly interested in decreasing their dietary intake of animal-based food products, due to health, sustainability, and ethical concerns. For this reason, the food industry is creating new products from plant-based ingredients that simulate many of the physicochemical and sensory attributes associated with animal-derived foods, including milk, eggs, and meat. An understanding of how the ingredient type, amount, and organization influence the desirable physicochemical, sensory, and nutritional attributes of these plant-based foods is required to achieve this goal. A potential problem with plant-based diets is that they lack key micronutrients, such as vitamin B_12_, vitamin D, calcium, and ω-3 fatty acids. The aim of this review is to present the science behind the creation of next-generation nutritionally fortified plant-based milk substitutes. These milk-like products may be formed by mechanically breaking down certain plant materials (including nuts, seeds, and legumes) to produce a dispersion of oil bodies and other colloidal matter in water, or by forming oil-in-water emulsions by homogenizing plant-based oils and emulsifiers with water. A brief overview of the formulation and fabrication of plant-based milks is given. The relationship between the optical properties, rheology, and stability of plant-based milks and their composition and structure is then covered. Approaches to fortify these products with micronutrients that may be missing from a plant-based diet are also highlighted. In conclusion, this article highlights how the knowledge of structural design principles can be used to facilitate the creation of higher quality and more sustainable plant-based food products.

## 1. Introduction

The modern food industry produces an impressive range of high-quality, safe, and tasty products for consumers, but it is increasingly putting a strain on our environment and leading to health problems [1,2]. As the world population grows and becomes wealthier, consumers are demanding a more Western-style diet, which often contains high levels of highly processed foods rich in fat, salt, and sugar [3]. As a result, incidences of diet-related chronic diseases, particularly diabetes and obesity, have risen in many countries [4]. Moreover, people are including higher amounts of animal-based products in their diet (meat, fish, eggs, and milk), which is having deleterious effects on the environment [1]. In particular, raising animals for food (including growing the feed required to sustain them) is often much more damaging to the environment than directly growing plant foods for humans, causing more pollution, increasing land utilization, depleting water resources, and generating more greenhouse gas emissions [4]. In addition, the consumption of high amounts of certain animal products is linked to adverse health outcomes, and causes major ethical concerns due to the confinement and slaughter of huge numbers of animals. Consequently, reducing the level of animal-based products consumed would be highly beneficial. Indeed, the results of a comprehensive study on sustainable diets by the EAT-Lancet commission [4] suggest that there should be a major reduction in the amount of animal foods consumed globally, which is in accordance with the recommendations of the 2030 Agenda for Sustainable Development of the United Nations’ Food and Agriculture Organization (FAO). The focus of this manuscript is the replacement of cow’s milk with plant-based alternatives. If these products are going to be successful, then it is important that they are safe, affordable, convenient, shelf-stable, and delicious, otherwise consumers will not include them in their diet. The main aim of this article is therefore to introduce the scientific principles required to create high-quality plant-based milk substitutes.

Plant-based milk substitutes have become increasingly popular over the past few years and numerous commercial products are already available, with some of the most popular being almond, coconut, flaxseed, oat, rice, and soy milks [5,6,7,8]. Plant-based milk substitutes are typically designed to have a fairly similar look, feel, taste, and shelf-life as cow’s milk so that they can be used in similar applications. In practice, however, each of them has its own unique properties that depend on the particular ingredients and unit operations utilized to create it. Some consumers find the difference in quality attributes between plant-based milk substitutes and cow’s milk to be unacceptable, which is holding back their more widespread adoption. Moreover, the functional attributes of plant-based milk substitutes are also different to those of cow’s milk, such as their stability in hot beverages (tea or coffee), their utilization during cooking, or their ability to be whipped into a foam. Many consumers would like to be able to use plant-based milk substitutes in the same applications that they use cow’s milk, so that they can simply substitute one product for another. The creation of good quality plant-based milk substitutes that have appropriate functional attributes depends on understanding the science behind their formation, properties, and performance.

In a recent review article, we focused on presenting the science underpinning the physicochemical and sensory attributes of plant-based milk substitutes [8]. In the current article, the main focus is on the fortification of plant-based milk substitutes with essential nutrients and nutraceuticals. Nevertheless, an overview of the physicochemical and sensory attributes of these products is also given for the sake of completeness.

## 2. Plant-Based Milk Production

The manufacturing processes utilized to fabricate plant-based milk substitutes should ideally produce a final product that has some similarities to cow’s milk in terms of their composition and structure [8]. In particular, the colloidal nature of cow’s milk plays a crucial role in producing its desirable quality attributes, since the fat globules and casein micelles contribute to its milky appearance and creamy texture [9]. The sugars and salts found in milk also play a key role in determining its flavor profile. Hence, plant-based products typically consist of colloidal fat and/or protein dispersed within an aqueous solution containing sugars and salts. In general, two main approaches have been developed to create this kind of product [8]: (i) Breaking down the natural structures of certain plant materials to release oil bodies or other colloidal matter [5], and (ii) the fabrication of simulated fat globules using plant-based ingredients [10].

### 2.1. Extracted Oil Bodies

Many edible plants develop specialized tissues (seeds) where lipids are stored as a source of energy and materials that can be utilized during biosynthesis [11]. The lipids are usually contained in oil bodies that are small colloidal particles (typically 0.2 to 2 μm) consisting of a triglyceride core coated by a phospholipid shell that has proteins embedded in it [12]. This kind of structure is similar to the fat globules in raw cow’s milk [13], and therefore would be expected to exhibit many of the same functional attributes, such as creating a creamy appearance and texture, in plant-based milk substitutes [8].

**Figure 1:**
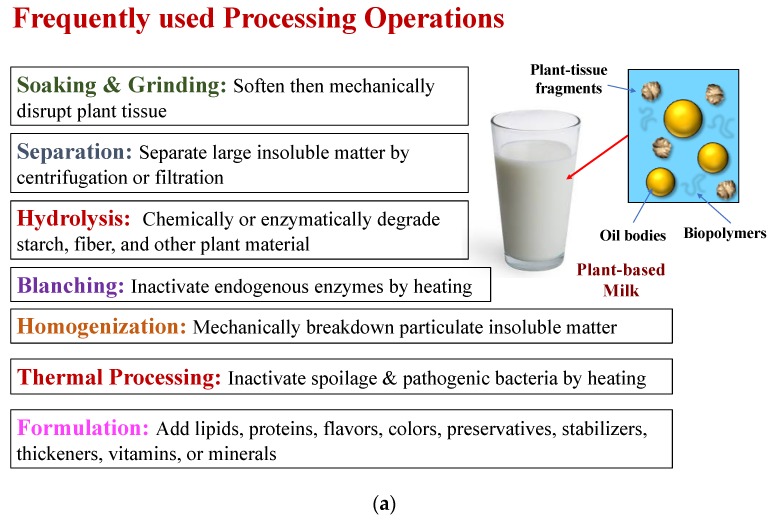

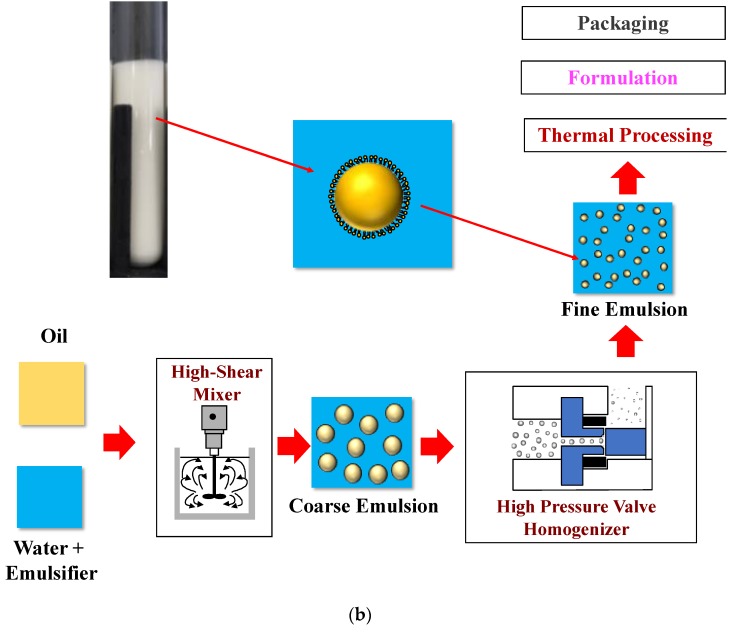
(**a**) Some commonly used processing operations that are utilized to produce plant-based milk substitutes. These processes need not be carried out in the order shown. Adapted from T. McHugh [17]. (**b**) Plant-based milk substitutes can also be produced by homogenizing plant-based oil and emulsifiers with water. Typically, a coarse emulsion is formed first, which is then passed through a homogenizer.

Plant seeds have different names depending on their biological origin. For instance, the seeds from legumes are referred to as beans, whereas those from trees are called nuts. Some common types of seeds used to create plant-based milk substitutes are almonds, cashews, coconuts, flaxseeds, and soybeans [5,14]. Pure oils (rather than oil bodies) can be extracted from these plant sources and used to form plant-based milk substitutes (see next section), but large quantities of organic solvents are often required for this purpose, which is costly and environmentally unfriendly. More environmentally friendly processing operations can be utilized to isolate intact oil bodies from plant seeds, such as soaking them in water to soften their structure, grinding them to release the oil bodies, fractionating them to remove undesirable plant debris, homogenizing them to reduce the size of the particles, and thermally processing them to deactivate enzymes and microbes [14,15,16,17]. Plant seeds naturally contain proteins, polysaccharides, sugars, and minerals that may by dissolved or dispersed in the water phase where they create a flavor profile and nutritional attributes that somewhat resemble those of cow’s milk. Some of the common processes used to create plant-based milks using this approach are highlighted in Figure 1a and discussed in more detail in a previous article [8]. This kind of product may be fortified with nutrients or nutraceuticals by blending them with the plant-based milk substitute during or after the manufacturing process, which is discussed in detail later.

Even though plant-based milk substitutes containing natural oil bodies have many compositional and structural similarities to cow’s milk, they cannot completely mimic its desirable physicochemical and sensory attributes. Cow’s milk has a relatively bland but distinctive flavor profile due to the unique combination of sugars, salts, and lipids it contains [18]. The aroma, taste, and mouthfeel of plant-based milk substitutes are different because they contain different kinds of molecules and structures. For instance, soy milks have been reported to have a characteristic beany flavor [5]. Many consumers find this undesirable because they do not like this kind of flavor profile or because it does not conform with their expectations of what milk should taste like.

### 2.2. Simulated Fat Globules

Plant-based milk substitutes with physical properties and sensory attributes fairly similar to cow’s milk can be produced from plant-based ingredients, such as plant-derived emulsifiers, oils, and others [10]. In this case, the plant-based ingredients are combined together to form an emulsion using a homogenization step (Figure 1b). The stability and properties of this type of colloidal dispersion depend strongly on the kind of ingredients and unit operations utilized during its production [19].

#### 2.2.1. Ingredients

*Oil*: The bulk oils used to prepare emulsions are isolated from a range of oil-rich plant materials, including coconut, corn, flaxseed, olive, palm, sesame, soybean, sunflower, and vegetable oils. These oils have different compositions, physical properties, chemical reactivities, flavor profiles, and nutritional attributes, which impact their application within plant-based milk substitutes. The fatty acid profile of oils, such as the ratio of saturated, monounsaturated, and polyunsaturated fats, impacts their nutritional profile. Liquid oils can be homogenized directly, whereas solid fats have to be melted first. The interfacial tension and viscosity of oils influence the sizes of the droplets generated inside a homogenizer, which affects the emulsion stability and quality [19]. Oil-soluble nutrients or nutraceuticals are typically dissolved within the oil phase prior to homogenization.

*Emulsifier*: Numerous plant-based emulsifiers are utilized by formulators to create emulsions, such as biosurfactants, phospholipids, proteins, and polysaccharides [20,21]. These are currently isolated from natural plant sources (such as soybeans, wheat, rice, oat, peas, flaxseed, or faba beans), but in the future, they may also be produced by cellular agriculture [22,23]. Plant-based emulsifiers differ appreciably in their capacity to form and stabilize emulsions. Typically, the smaller and more surface-active the emulsifier, the more effective it is at generating fine droplets inside a homogenizer. This is why smaller droplets can be produced using small biosurfactants (such as quillaja saponins) than large biopolymers (such as gum arabic) when the same homogenization conditions are used. Conversely, plant-based emulsifiers that produce thicker interfacial layers often provide better emulsion stability because they lead to intense steric repulsive forces that inhibit droplet aggregation. This is why emulsions that are more stable to environmental stresses can be produced from large biopolymers (such as gum arabic) than small biopolymers (such as pea protein). The advantages and disadvantages of different kinds of plant-based emulsifiers for forming and stabilizing the oil droplets in plant-based milk substitutes has been discussed recently [8].

*Additives*: A variety of other ingredients can be incorporated into plant-based milk substitutes to improve their functional properties [20,21]. Thickening agents can be used to create desirable textural or mouthfeel attributes, or to inhibit the creaming of lipid droplets or the sedimentation of insoluble matter. Numerous kinds of biopolymers can be employed as thickening agents, which vary in their molecular, physical, functional, and biological properties. For a fully plant-based milk, these biopolymers should be derived entirely from plants, e.g., starch, pectin, corn fiber, or locust bean gum. In some products, however, it may be acceptable to use other non-animal-based biopolymers, such as those isolated from seaweed (e.g., alginate and carrageenan) or obtained by microbial fermentation (e.g., xanthan gum). Anionic biopolymers, such as carrageenan or pectin, may also be used in milk-like products to inhibit the flocculation of protein-coated droplets. They do this by adsorbing to cationic patches on the surfaces of the oil droplets, thereby creating a thick coating that stops the droplets from coming close together [24].

A range of other additives may also be incorporated to enhance the quality attributes, nutritional profile, or safety of plant-based milk substitutes, including minerals, vitamins, nutraceuticals, colors, flavors, or preservatives. It is important that these ingredients are carefully selected so that they do not promote any undesirable changes in the quality attributes or stability of the end product. A number of nutrition scientists have criticized plant-based milk substitutes based on the fact that they do not contain all of the essential nutrients found in cow’s milk, such as high-quality proteins, minerals (such as calcium), and vitamins (such as vitamins A, D, E, and B_12_). For this reason, many manufacturers of plant-based milk substitutes are incorporating these ingredients into their products. Typically, non-polar nutritional components are added to the oil phase before homogenization, whereas polar ones are added to the water phase before or after homogenization. As an example, calcium carbonate and tricalcium phosphate are being added as a source of calcium. These colloidal forms of calcium are selected because they are insoluble and so do not promote aggregation of the anionic proteins in plant-based milk substitutes due to ion binding and electrostatic screening effects. The calcium is present in colloidal particles that can be dispersed throughout the product but shaking may still be required to avoid their sedimentation.

#### 2.2.2. Manufacturing Operations

The manufacture of plant-based milk substitutes using this approach requires a homogenization step to transform the oil and water phases into an oil-in-water emulsion (Figure 1b) [25]. First, the oil-soluble ingredients are dissolved within the oil phase, while the water-soluble ingredients (including a hydrophilic emulsifier) are dissolved into the water phase. Second, an emulsion pre-mix is produced by blending the oil phase and water phase together. Third, a fine emulsion is produced by passing the emulsion pre-mix through a mechanical device (a “homogenizer”) that further breaks down the oil droplets. A number of homogenizers can be used for this purpose, including colloid mills, high-pressure valve homogenizers, microfluidizers, and sonicators. The same mechanical devices are typically used in the dairy industry to convert raw milk into homogenized milk. There are benefits and drawbacks to each type of homogenizer, which have been discussed in detail elsewhere [19]. In particular, they vary in their purchasing, running, and maintenance costs; their throughput; the viscosity of samples they can handle; their robustness; and the size of the droplets they can produce. After formation, plant-based milk substitutes typically undergo some form of heating step to extend their shelf-life and ensure they are safe to consume. This thermal processing step is designed to deactivate any enzymes or microbes that may cause spoilage or health problems, while still maintaining high product quality. The ability of plant-based milk substitutes to remain stable after thermal processing depends on the type of emulsifier used, with natural surfactants or polysaccharides giving better stability than proteins [20]. The impact of the emulsifier type on the thermal stability of plant-based milk substitutes has been discussed in more detail in a recent review article [8].

## 3. Structural Basis of Physicochemical Properties

Plant-based milk substitutes should have physical, functional, and sensory properties that consumers find desirable, which means that manufacturers must understand the major factors that govern their properties and performance. Plant-based milk substitutes are complex colloidal dispersions and so many of their properties are determined by the nature of the polymers and particles they contain, as well as how they interact with each other.

### 3.1. Visual Attributes

The first impression a consumer has of a plant-based milk is usually its appearance. Typically, this kind of product should have visual attributes fairly similar to cow’s milk, i.e., a uniform milky white. However, the expected appearance also depends on the precise characteristics of the product. For example, consumers may accept a more brownish tint to a nut-based milk. Obviously, flavored products, such as chocolate, banana, or strawberry milks, are expected to have distinctive colors, such as brown, yellow, or pink.

#### 3.1.1. Physical Basis of Visual Attributes

**Figure 2:**
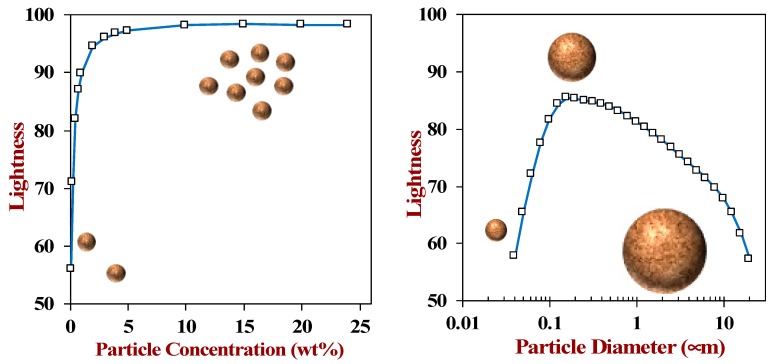
The lightness of a plant-based milk substitute is dependent on the size and concentration of any particulate matter that scatters light. The theoretical predictions of lightness were made by the author using light scattering theory for oil droplets dispersed in water.

*Scattering*: The characteristic creamy look of plant-based milk substitutes is a result of the scattering of visible light waves by the colloidal particles they contain, such as lipid droplets, oil bodies, protein particles, or plant fragments [19]. Light waves are scattered by colloidal particles when they have a different refractive index to the liquid that surrounds them, which is the case for particles comprised of fat, protein, or carbohydrate [26]. The visual creaminess of a product is governed by the degree of light scattering that occurs, which is mainly governed by the refractive index contrast, dimensions, and concentration of the scattering particles [27]. Light scattering becomes more intense as the number of colloidal particles in a plant-based milk is increased. The intensity of light scattering also depends on the dimensions of the colloidal particles compared to the wavelength of light, with a maximum value occurring when the particles have diameters around 200 nm (Figure 2). The optical properties of plant-based milk substitutes can therefore be optimized by carefully manipulating the concentration and dimensions of the lipid droplets or oil bodies they contain. Alternatively, the visual creaminess may be increased by adding other plant-based ingredients that scatter light strongly, such as protein particles or plant tissue fragments, which may be an advantage for low-fat or low-calorie products. In practice, experimental measurements on plant-based milk products do not show a strong correlation between their lightness and particle size, although smaller particles do seem to lead to a somewhat lighter product (Table 1). This may be because these commercial products contain a wide range of different components that may scatter or absorb light.

*Absorption*: The characteristic color of plant-based milk substitutes is mainly due to the selective absorption of visible light waves by any pigments [27]. These pigments may be naturally present within the plant materials used to create the milks, such as chlorophyll, anthocyanins, or carotenoids. Alternatively, they may arise as a result of the processing operations used to create the products, e.g., the brownish Maillard reaction products formed when proteins and reducing sugars are heated together. Natural pigments may be deliberately incorporated into plant-based milk substitutes to obtain appearances that correspond to consumer expectations, such as brown (chocolate), yellow (banana), or pink (strawberry) colors for flavored milks. Alternatively, some of the nutraceuticals that may be incorporated into plant-based milk substitutes may be naturally pigmented themselves, and thereby alter the color of the final products, such as curcumin (orange) or carotenoids (red, orange, or yellow). Consequently, a manufacturer may have to take this into account when designing a nutritionally fortified product.

#### 3.1.2. Examples of Plant-Based Milk Appearance

Colorimetry has been used to characterize the appearance of plant-based milk substitutes, with the L*a*b* color space being commonly used to quantify their optical properties. Here, L* represents the lightness (0 to 100 = dark to light), a* represents the redness (+)/greenness (−), and b* represents the yellowness (+)/blueness (−). Measurements of the color coordinates of commercial soy milks have indicated that their optical properties vary considerably from product to product: L* = 62 to 81; a* = −3 to +3; b* = +7 to +23 [29]. Colorimetry measurements on numerous commercial plant-based milk substitutes (including almond, cashew, hazelnut, hemp, oat, rice, and soy milks) have shown that their whiteness index (calculated from the L*a*b* values) may vary quite considerably (Table 1), ranging from around 52 to 76, which is less than the value of 82 reported for cow’s milk [28]. The color variations seen in plant-based milk are a result of differences in their compositions and structures, which arises due to the different ingredients and unit operations used to create them. Consequently, the degree of light scattering and absorption is different for different products. Plant-based milk substitutes prepared using lentil proteins as emulsifiers were found to have a slightly pinkish color, which was attributed to some pigments arising from the lentils [30]. A thermal treatment (85 °C) of these products was shown to reduce their pink color, which was attributed to the thermal degradation of anthocyanins from the lentils. The incorporation of oil into these products increased their lightness from around 46 to 77, presumably due to the generation of small protein-coated lipid droplets that scattered light. The effective of high-pressure homogenization on the appearance of plant-based milk substitutes has also been investigated [31,32]. Passing the milks through a high-pressure homogenizer could be used to increase their lightness because it reduced the particle size, thereby leading to more intense light scattering. Other researchers have examined the impact of adding plant-based milk substitutes to hot black coffee on the appearance of the final drink [33,34]. The lightness of the final coffee depended on the size and concentration of the lipid droplets in the plant-based milk. Recent studies have shown that fortifying plant-based milk substitutes with curcumin makes them turn a yellowy-orange color [35,36], which would have to be considered when marketing the products.

### 3.2. Textural Attributes

#### 3.2.1. Physical Basis of Textural Attributes

Plant-based milk substitutes are colloidal dispersions consisting of particles (oil bodies, lipid droplets, protein aggregates) and biopolymers (proteins or polysaccharides) dispersed within a watery fluid that contains sugars and salts. To a first approximation, the textural attributes of plant-based milk substitutes can therefore be predicted using the theoretical equations developed to model colloidal dispersions [19]. The shear viscosity of a dilute suspension of rigid spherical particles (*η*) is given by:

(1) $$\eta =\eta_{1}\left(1+2.5\phi \right).$$

In this equation, often referred to as the Einstein equation, *ϕ* is the volume fraction of the colloidal particles and *η*_1_ is the viscosity of the fluid surrounding the particles. The viscosities of concentrated suspensions are higher than the values predicted by the Einstein equation due to interactions between the particles. In this case, semi-empirical equations are typically more suitable for describing the dependence of the shear viscosity on particle concentration, such as the one show below [19]:

(2) $$\eta =\eta_{1}{\left(1+\frac{\phi }{\phi_{C}}\right)}^{-2}.$$

**Figure 3:**
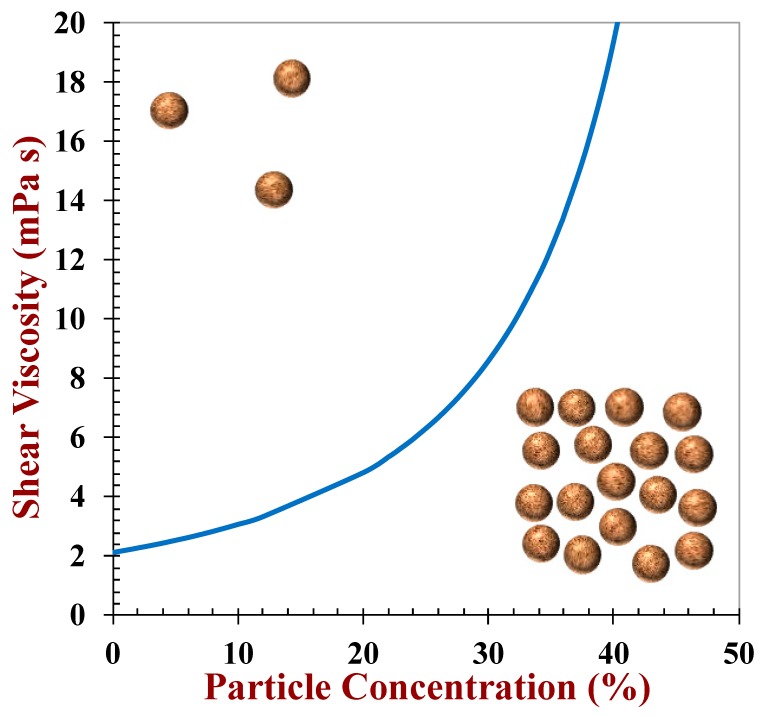
The shear viscosity of plant-based milks depends on the concentration of particles they contain, such as oil bodies or fat droplets. The viscosity may also be increased by adding thickening agents. The theoretical predictions were made by the author using effective medium theory for oil droplets suspended in water.

In this equation, *ϕ*_*C*_ represents the volume fraction where the particles are so tightly packed that the overall system attains some elastic properties, which typically occurs when the particles occupy around 65% of the volume (i.e., *ϕ*_*C*_ ≈ 0.65). This equation predicts that the shear viscosity initially increases fairly gradually as the particle concentration is raised, but then it increases very steeply when the particles become more closely packed (Figure 3). This semi-empirical equation accounts for the fact that plant-based milk substitutes, which contain relatively low amounts of fat droplets or oil bodies (<5%), typically have relatively low viscosities (provided they do not contain thickening agents).

#### 3.2.2. Examples of Plant-Based Milk Texture

Plant-based milk substitutes are typically formulated so that they have textural attributes that closely resemble those of conventional milk products, such as milk or cream. As predicted by Equation (2), the viscosities of cow’s milk products increase as their fat content increases, being 2.2, 2.6, 4.9, 8.8, and 22.3 mPa s for skim milk (0.5% fat), whole milk (3.3% fat), half-and-half (14% fat), light cream (24% fat), and heavy cream (40% fat), respectively [8]. Cow’s milk products tend to exhibit ideal (Newtonian) flow behavior at low fat contents (<20%) but may exhibit some shear thinning at higher fat contents [37]. Manufacturers of plant-based products often try to mimic the rheology of these cow’s milk products by controlling the amount of oil bodies or fat droplets present, or by adding plant-based thickening agents.

The textural attributes of some commercial plant-based milk products have been reported in the literature (Table 1). These instrumental measurements indicate that they are typically low viscosity fluids with rheological properties fairly similar to those of cow’s milk products. Other researchers have also shown that plant-based milk substitutes have shear viscosities that are fairly similar to those of cow’s milk products, e.g., 2.2 to 3.4 mPa s for lentil milk [30], 1.2 to 9.9 mPa s for soy milk [29], 3.9 mPa s for almond milk [38], and 1.6 mPa s for hazelnut milk [39]. Differences in the rheology of plant-based milk products are mainly due to variations in the amount of oil bodies, fat droplets, or other colloidal matter they contain, as well as in the presence of any thickening agents. The molar mass, conformation, concentration, and molecular interactions of a thickening agent all influence its ability to increase the viscosity of an aqueous solution, as well as its shear thinning behavior, and so it is critical to choose the most suitable one. In some cases, appropriate textural properties can be achieved using a combination of thickening agents. As well as altering the textural attributes of the final product, a thickening agent may also be added to inhibit gravitational separation of the colloidal particles, such as the creaming of oil bodies or fat droplets, or sedimentation of protein particles or plant fragments.

Some plant-based milk substitutes contain relatively high levels of soluble plant-based polysaccharides that thicken the aqueous phase because of their extended conformations in water. These polysaccharides may arise from the plant materials used to prepare the milk (such as almonds, coconuts, oats, or soybeans) or they may be added as functional ingredients (such as guar or locust bean gums). The presence of these polysaccharides modulates the mouthfeel and textural attributes of plant-based milk substitutes, as well as increasing their resistance to separation due to gravitational forces. For instance, the increase in viscosity reduces the rate at which fat droplets or oil bodies rise, or that insoluble plant material falls. The presence of extended polysaccharides in the aqueous phase of plant-based milk substitutes may lead to shear thinning behavior, where the viscosity decreases as the shear rate increases. For an ideal fluid, the flow index is equal to one, but for a shear-thinning fluid it is less than one. As seen in Table 1, some plant-based milk substitutes exhibit ideal behavior, whereas others exhibit pronounced shear thinning behavior. The viscosity of these shear-thinning milks depends on the shear rate applied to them. This phenomenon has also been reported for oat milks, where there is an appreciable decrease in the apparent shear viscosity with an increasing shear rate [40]. For instance, the viscosity of oat milk (20° Brix, 20 °C) decreased from 1400 to 74 mPa s when the shear rate was increased from 1 to 100 s^−1^. The processability, functional properties, and sensory attributes of plant-based milk substitutes are likely to depend on how their viscosity changes with the shear rate, and so it may be important for manufacturers to control this.

### 3.3. Stability

The ability of plant-based milk substitutes to resist changes in their desirable quality attributes throughout storage or when they are exposed to specific environmental conditions (such as thermal processing or addition to coffee) is an important characteristic for commercial applications. For this reason, it is critical to develop a strong appreciation of the factors impacting the stability of plant-based milk substitutes. The stability of a specific commercial product is dependent on the kind of particles within it (e.g., oil bodies, fat droplets, or plant fragments), the nature of the aqueous solution (e.g., pH, mineral composition, sugar content, viscosity, and density), as well as any mechanical forces and temperature changes the product experiences throughout its lifetime.

**Figure 4:**
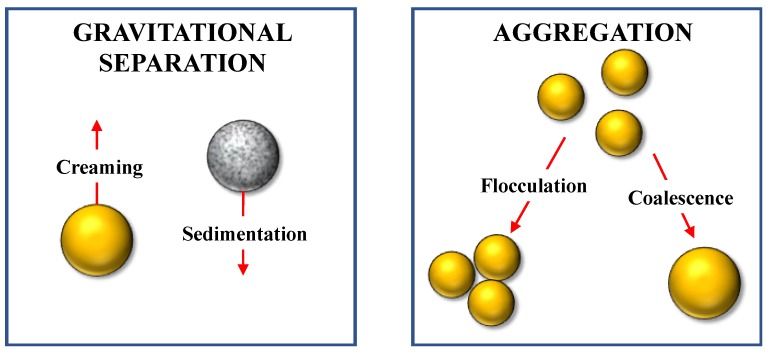
The oil droplets, oil bodies, or plant tissue fragments in plant-based milk substitutes can separate or aggregate, thereby reducing product stability.

In principle, plant-based milk substitutes may destabilize as a result of various physical processes, such as flocculation, coalescence, partial coalescence, oiling off, creaming, and sedimentation (Figure 4) [19]. Plant-based milk substitutes can also destabilize due to various chemical or biochemical processes, including oxidation or hydrolysis. Moreover, they may become unstable or unsafe as a result of microbiological processes, including the growth of bacteria, yeasts, or molds. Knowledge of the different mechanisms responsible for the breakdown of plant-based milk substitutes is critical for designing products with sufficiently long shelf-lives and with good functional performances in different applications.

#### 3.3.1. Physical Instability

*Gravity-Induced Separation*: Plant-based milk substitutes contain particulate matter that has a density that is different from that of the watery fluids they are dispersed in, which leads to a gravitational pull on them [19]. Particulate matter that is less dense than water, such as oil bodies or fat droplets, tends to rise (“cream”), whereas particulate matter with a higher density, such as plant cell fragments, starch granules protein aggregates, or calcium carbonate particles, tends to sink (sediment).

The velocity (*v*) a rigid spherical particle travels upwards in an ideal liquid as a result of gravity is given by Stokes’ law for dilute systems:

(3) $$v=-\frac{2gD^{2}\left(\rho_{2}-\rho_{1}\right)}{18\eta_{1}}.$$

In this equation, *D* is the radius of the particles, *ρ*_2_ is the density of the particles, *ρ*_1_ is the density of the surrounding liquid, *η*_1_ is the viscosity of the surrounding liquid, and *g* is the acceleration due to gravity. Although the particles in plant-based milk substitutes are not strictly rigid spheres that do not interact with each other, Stokes’ law still provides some valuable insights. It shows that the velocity a particle moves upwards with decreases as the particle diameter decreases, the density contrast decreases, or the viscosity of the surrounding fluid increases. For relatively light particles (*ρ*_2_ < *ρ*_1_), the creaming velocity has a positive sign, meaning they tend to rise. For relatively heavy particles (*ρ*_2_ > *ρ*_1_), the creaming velocity has a negative sign, meaning they tend to sink.

**Figure 5:**
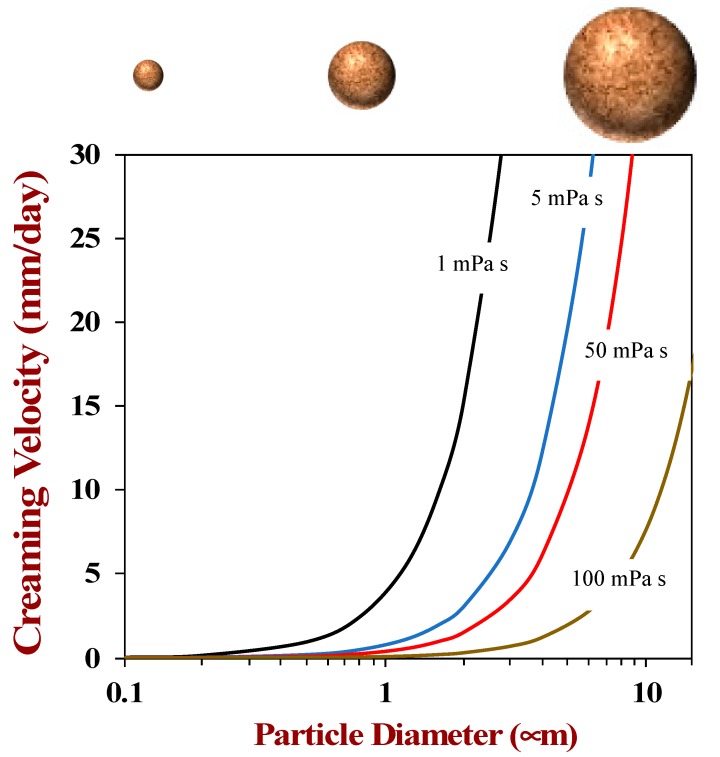
The creaming stability of plant-based milks depends on the size of the particulate matter they contain. The theoretical predictions were made by the author using Stoke’ law for oil droplets of different sizes suspended in aqueous solutions with different viscosities.

In general, Stokes’ law shows that the stability of plant-based milk substitutes to gravitational separation may be improved by reducing the particle diameter, increasing the aqueous phase viscosity, or reducing the density contrast. As an example, Stokes’ law was used to predict the impact of the particle size and aqueous phase viscosity on the creaming stability of the fat droplets in a model plant-based milk (Figure 5). The creaming rate becomes relatively fast once the diameter of the particles exceeds about 500 nm, which shows why it is important to have relatively small particles in plant-based milk products. This could be achieved by selecting plant species that naturally contain relatively small oil bodies or by effectively homogenizing any oil phase added so as to produce small fat droplets. In practice, the size of the particles in most plant-based milk substitutes is relatively large, with the mean diameter (D_32_) being around a micrometer. Typically, there is a wide range of particle sizes in commercial products, which means that some particles (the large ones) are much more susceptible to gravitational separation than others. The rate of creaming or sedimentation can be inhibited by adding thickening agents to increase the viscosity of the aqueous phase, e.g., gellan gum, guar gum, or locust bean gum. Nevertheless, one cannot add too much thickening agent, or it will adversely impact the texture, pourability, and mouthfeel of the product.

*Aggregation*: The aggregation of the particulate matter in plant-based milk substitutes is a serious quality defect in some applications (Figure 4). An everyday example of this instability mechanism is the “feathering” that occurs when some plant-based milk substitutes are added to hot coffee. This quality defect is the result of the aggregation of the oil bodies, protein-coated fat droplets, protein particles, and/or plant cell fragments in plant-based milk substitutes due to an alteration in the colloidal interactions amongst them. The most likely origin of this effect is the change in the electrostatic repulsion between protein-based particles or polymers in the system. Coffee is mildly acidic (pH ≈ 5) and so it has a pH that is close to the isoelectric point (pI) of many plant proteins. As a result, the proteins lose most of their electrical charge, which reduces the magnitude of the electrostatic repulsive forces that normally prevent them from coming into close contact and aggregating. The aggregation of oil bodies or fat droplets leads to the formation of large diffuse clumps that give an undesirable feathery appearance on the surface of the product. Conversely, the aggregation of the protein particles leads to the formation of a sediment on the bottom of the product. In principle, this problem may be overcome in a number of ways, which are more or less economically viable: (i) Change the nature of the plant-based emulsifier used to stabilize the system, (ii) include an anionic plant-based polysaccharide that interacts with the proteins and stops them from aggregating, (iii) include a buffering agent that prevents the pH from getting too low, and (iv) include a chelating agent that binds any free calcium ions that may be released when the pH is reduced.

The shelf-life of plant-based milk substitutes is strongly dependent on the aggregation stability of the colloidal particles they contain [19]. Aggregation may lead to a number of undesirable changes in the perceived quality of plant-based milk substitutes: (i) Gravitational separation may lead to the formation of an undesirable layer of particles at the top or bottom of the product, (ii) some aggregates may become so large that they are visible within the product, and (iii) some aggregates may become so large that they cause the mouthfeel to become gritty or grainy. The tendency for particle aggregation to occur is governed by the relative magnitude of the various kinds of attractive and repulsive colloidal interactions operating in the system, such as van der Waals, steric, hydrophobic, depletion, bridging, and electrostatic interactions [19]. Aggregation will tend to occur when the attractive interactions dominate the repulsive ones. A good understanding of the major factors that influence the various types of colloidal interactions is therefore important for enhancing the aggregation stability of plant-based milk substitutes. Some of the ways of increasing the stability of plant-based milk substitutes are highlighted in Table 2, including increasing the steric or electrostatic repulsion, or decreasing the hydrophobic, depletion, or bridging attraction [8]. The ingredient formulation or processing operations used to produce plant-based milk substitutes can often be controlled to ensure that they have good resistance to aggregation. For instance, reducing the size of the colloidal particles often decreases the tendency for aggregation to occur. In addition, controlling the pH, as well as the type and level of mineral ions and polymers present, can also have a major influence on the stability of plant-based milk substitutes.

In general, the colloidal particles within plant-based milk substitutes may aggregate as a result of flocculation (two or more particles stick together) or coalescence (two or more particles merge together) depending on the nature of their surface and core properties (Figure 4). Particles that are solid or have “rigid” surfaces will only tend to flocculate, where those that are fluid and have “soft” surfaces may both flocculate and coalescence. The most likely particles to coalesce in plant-based milk substitutes are oil droplets or oil bodies, which have a fluid oil core. If many of these particles coalesce with each other, then an unsightly layer of oil may eventually form on the surface of the product.

#### 3.3.2. Chemical Instability

It is also important to consider the potential chemical instability of any nutrients or nutraceuticals added to plant-based milk substitutes. Many of these bioactive compounds are chemically labile molecules that tend to degrade throughout storage, including ω-3 fatty acids [41,42], carotenoids [43,44], curcumin [45], quercetin [46], resveratrol [47], and oil-soluble vitamins [48]. Consequently, it is important to elucidate the reaction mechanisms involved and to identify the major factors impacting their degradation rate (such as pH, metal ions, temperature, oxygen, and light exposure). Appropriate strategies can then be employed to inhibit their chemical degradation during the storage of plant-based milk substitutes.

#### 3.3.3. Examples of Instability in Plant-Based Milk Substitutes

A number of studies have shown that plant-based milk substitutes are unstable under certain conditions. For instance, soy lecithin was used to partially replace a milk protein (sodium caseinate) in a model plant-based milk [49]. When the milk protein-coated droplets were added to coffee, the system exhibited extensive particle aggregation and creaming, presumably due to a decrease in the electrostatic repulsive forces acting amongst the droplets. This phenomenon occurred because coffee is fairly acidic (pH 5) and so the negative charge on the protein molecules in the milk decreases when the pH approaches their isoelectric point. A similar phenomenon would be expected for plant protein-coated fat droplets [41] but not for saponin-coated ones, which remain stable after being incorporated into coffee because they maintain a strong negative charge [50].

The stability of various commercial plant-based milk products to gravitational separation during storage is compared in Table 1 [28]. Appreciable variations were observed in the resistance of these products to separation, being over 40-fold higher for the most stable product compared to the least stable one. These differences may be a result of differences in the particle size and density, as well as differences in the aqueous phase viscosity. One would expect products with higher viscosities and smaller particle sizes to be the most stable based on Stokes’ law. The influence of different factors on the physical stability of coconut milks has recently been reviewed [51]. Coconut milk was reported to exhibit extensive aggregation and creaming close to the coconut proteins’ isoelectric point, as well as at high salt levels, which was attributed to a decrease in the electrostatic repulsive forces in the system. The coconut milk was also reported to be unstable to aggregation and creaming after heating to temperatures above the thermal denaturation temperature of the coconut proteins. This effect is a result of an increase in the hydrophobic attraction amongst the coconut protein-coated fat droplets or oil bodies. The stability of coconut milk could be increased by adding emulsifiers and homogenizing to reduce the particle size. Other types of plant-based milk substitutes have also been shown to be unstable to aggregation and creaming when the pH is around the protein’s isoelectric point, when the mineral levels are high, or when they are subjected to heating due to similar reasons, e.g., those stabilized by soy proteins [52,53], lentil, pea, or faba bean proteins [41]. The impact of protein type and location on the oxidative stability of plant-based emulsions containing flaxseed oil has been investigated [42]. This study showed that non-adsorbed plant-based proteins increased the stability of emulsified ω-3 fatty acids to oxidation, presumably by binding transition metal ions and keeping them away from the oil droplet surfaces.

### 3.4. Sensory Properties

If plant-based milk substitutes are going be a successful alternative to bovine milk, then they will have to be desirable to consumers [54,55]. Many people have grown up consuming cow’s milk and so are used to its subtle flavor profile and creamy mouthfeel [56]. The desirable look, aroma, taste, and mouthfeel of cow’s milk are strongly dependent on the presence of milk fat globules [57]. The characteristic creamy appearance of cow’s milk is a result of scattering by these fat globules. The creamy texture and mouthfeel are due to the ability of the fat globules to modulate the fluid flow profile and lubricate the tongue. The buttery flavor profile is due to specific non-polar flavor molecules solubilized within the fat globules. Ideally, plant-based milk substitutes should be designed to mimic these desirable sensory properties.

Many plant-based milk substitutes have their own unique flavor profiles, which depend on the volatiles arising from their ingredients, such as “almond”, “bean”, “coconut”, “nut”, or “oat” flavors [54]. In some cases, these flavors may be perceived as a desirable quality attribute, but in other cases they are undesirable and may have to be removed or masked. Some plant-based milk substitutes also contain particulate matter, such as plant cell debris or colloidal calcium, that gives an unpleasant grittiness or chalkiness in the mouth. In some circumstances, homogenization can be used to break down the size of the particular matter, thereby reducing its adverse mouthfeel.

Recently, the sensory attributes of several commercial plant-based milk substitutes were compared in terms of their appearance, aroma, flavor, mouthfeel, and overall liking [30]. The overall liking of the plant-based milk substitutes was ranked in descending order as: Oat ≈ rice ≈ almond ≈ soy ≈ lentil > hemp. The sensory attributes (color, flavor, and mouthfeel) of two plant-based milk substitutes (almond and soy) were compared with those of cow’s milk [58]. The consumers ranked soy milk as having a significantly lower overall liking than cow’s milk, but almond milk had a fairly similar liking to cow’s milk.

## 4. Nutritional Fortification

**Figure 6:**
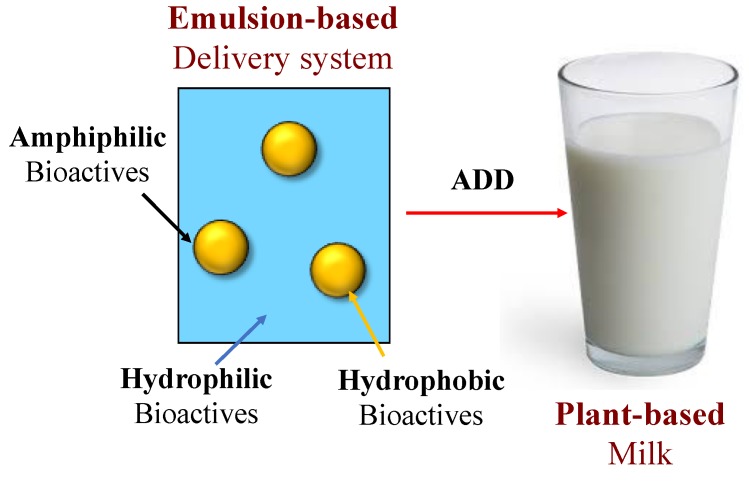
Bioactive agents with different polarities can be added to plant-based milks via emulsion-based delivery systems.

As mentioned earlier, an increasing number of consumers are replacing animal products in their diets, such as meat, egg, and milk, with plant-based ones due to ethical, health, and environmental reasons [5]. There are, however, some nutritional concerns associated with reducing the levels of animal products in the human diet [59]. In particular, plant-based diets are often deficient in important macronutrients and micronutrients, including high-quality proteins, omega-3 fatty acids, vitamin B_12_, vitamin D, iron, calcium, and iodine [60,61]. Over time, deficiencies in these nutrients could lead to health problems, particularly in infants and the elderly [60,62]. Consequently, it would be advantageous to supplement plant-based diets with these critical nutrients so as to avoid these deficiencies. Moreover, there may be additional health benefits associated with fortifying plant-based foods with nutraceuticals, such as carotenoids, curcuminoids, and polyphenols [63,64]. Plant-based milk substitutes are an ideal vehicle for introducing essential nutrients and nutraceuticals into the human diet. They can easily be incorporated into a person’s daily routine, e.g., as a beverage, creamer, or as part of a breakfast. They are colloidal dispersions that can be fortified with bioactive ingredients with different molecular polarities: Hydrophilic, hydrophobic, and amphiphilic (Figure 6). Third, they can be designed to increase the bioavailability of the encapsulated bioactive agents. Nevertheless, they must be carefully designed so that the bioactive ingredients remain stable throughout the shelf-life of the product, do not adversely affect product quality, and are highly bioavailable after ingestion. In the next section, we discuss the main factors affecting the incorporation of bioactive agents into plant-based milk substitutes, as well as those impacting their bioavailability. The main focus will be on hydrophobic bioactive agents (such as oil-soluble vitamins and nutraceuticals), since these are usually the ones that are most challenging to introduce into plant-based milk substitutes and have the lowest bioavailability.

### 4.1. Incorporation of Bioactive Agents into Plant-Based Milk Substitutes

There are a number of different approaches that can be used to fortify plant-based milk substitutes, which depend on the approach used to formulate them, as well as the physicochemical characteristics of the bioactive agents [8]. If a plant-based milk is formed by homogenization of bulk oil and water phases, then hydrophobic bioactives can simply be dissolved in the oil phase prior to emulsion formation, whereas hydrophilic ones can be added either before or after emulsification [65,66]. Recently, it has been reported that vitamin D has been incorporated into pea protein-stabilized oil-in-water nanoemulsions using this method [67]. This study showed that the cellular uptake of this oil-soluble vitamin (determined using Caco-2 cells as model epithelium cells) was increased 2.5-fold when the mean particle diameter was decreased from 350 to 233 nm, suggesting that smaller droplets were more effective at delivering the vitamin. In another study on plant-based emulsions stabilized by pea proteins, it was shown that the nature of the oil phase composition impacted the bioaccessibility of the oil-soluble vitamins [68]. In particular, monounsaturated oils (such as corn oil) gave a higher bioaccessibility than polyunsaturated oils (such as flaxseed oil). Other researchers have shown that oil-soluble vitamins can be incorporated into emulsions stabilized by other kinds of plant-based emulsifiers, such as soy protein [69] and soy lecithin [70].

If a plant-based milk is formed by breaking down the normal cellular structure of plant materials, then different approaches are required. Hydrophilic bioactives can often be simply dissolved or dispersed within the aqueous phase of this kind of plant-based milk. Nevertheless, special precautions may be required for some bioactive agents. For instance, calcium usually has to be introduced in a colloidal form (small solid particles), so as to avoid aggregation of any anionic constituents (such as proteins) in the milk promoted by free calcium ions (Ca^2+^). Hydrophobic bioactives can be introduced into preformed plant-based milk substitutes in various ways [71]. First, it may simply be possible to homogenize a bioactive-fortified oil phase with the plant-based milk to form bioactive-loaded oil droplets. These oil droplets will be stabilized by any surface-active ingredients naturally present in the aqueous phase of the plant-based milk substitutes, such as proteins or phospholipids. Second, it is possible to create a separate plant-based emulsion containing the hydrophobic bioactive agents dissolved in the oil phase [65,66], and then mix this with the plant-based milk. In this case, the hydrophobic bioactives may diffuse through the aqueous phase so that they are distributed throughout all of the oil droplets in the system. Third, for some hydrophobic bioactives, a pH-shift method can be used to load plant-based milk substitutes. For instance, in a recent study, we showed that a hydrophobic nutraceutical (curcumin) could be loaded into commercial plant-based milk substitutes (soymilk) using a pH-shift method [35,36]. The curcumin is highly soluble in water under strongly alkaline conditions (pH 12) but insoluble in water under neutral or acidic conditions (pH ≤ 7). Consequently, an alkaline curcumin solution can be mixed with a slightly acidic plant-based milk, which causes the curcumin to move from the aqueous phase into the oil bodies. Nevertheless, this latter method is only applicable to a limited range of bioactive agents that are highly soluble under one set of solution conditions but not under another.

The development of emulsion-based delivery systems is one of the most versatile for loading hydrophobic bioactive agents into preformed plant-based milk substitutes [65,66]. Previous studies have shown that the composition and structure of emulsion-based delivery systems has a major impact on the stability and bioavailability of encapsulated hydrophobic bioactive agents [66]. In particular, the oil phase composition, oil droplet size, and emulsifier type have been shown to have a major impact on the bioaccessibility, transformation, and/or absorption of hydrophobic bioactives [66,72]. Consequently, their properties can be carefully designed to produce plant-based milk substitutes in which the encapsulated bioactives have good stability during storage but are released in a highly bioavailable form within the gastrointestinal tract after ingestion. In the following section, the effect of the oil droplet size, composition, and surface properties on the bioavailability of hydrophobic bioactives is shown. Initially, however, an overview of the main factors impacting the bioavailability of hydrophobic bioactives is given.

### 4.2. Bioavailability

**Figure 7:**
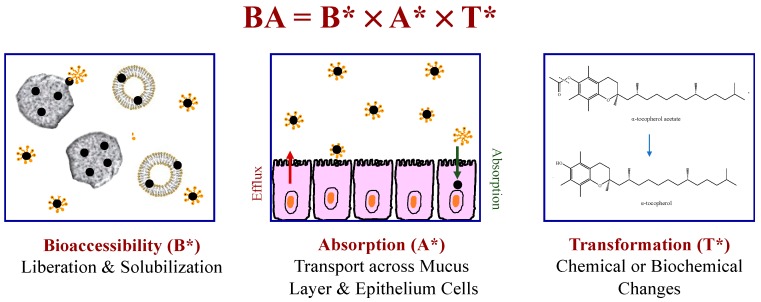
The oral bioavailability of a hydrophobic bioactive agent depends on liberation, solubilization, transport, transformation, and absorption processes.

*Bioavailability* can be conveniently defined as the fraction of an ingested micronutrient that ends up in the systemic circulation [73]. A number of factors impact the overall bioavailability (*F*) of bioactive agents: *F* = *F*_B_ × *F*_A_ × *F*_T_ (Figure 7). Here, *F*_B_ is the fraction liberated from the food matrix into the GIT fluids to become bioaccessible. *F*_T_ is the fraction that remains in a bioactive form after any chemical or biochemical transformations have occurred. *F*_A_ is the fraction of the bioaccessible bioactive agent that is absorbed by the epithelium cells. The major physicochemical and physiological processes determining these parameters have been identified [74,75,76,77].

*Bioaccessibility* (*F*_B_): First, the bioactive substances must be liberated from the food matrix and then solubilized within the intestinal fluids. Hydrophilic bioactives (like calcium ions and vitamin B_12_) are often dissolved or dispersed within the aqueous intestinal fluids, provided they do not strongly interact with other food or GIT components. Hydrophobic bioactives (like vitamin D and ω-3 fatty acids) typically have to be incorporated into mixed micelles within the small intestine in order to be solubilized. These mixed micelles consist of bile salts and phospholipids secreted by the body, as well as lipid digestion products (monoacylglycerols and free fatty acids).

*Transformations* (*F*_T_): A bioactive substance may undergo chemical and/or biochemical transformation as it passes through the GIT due to the change in its molecular environment and exposure to digestive and metabolic enzymes. These changes may alter its bioactivity, and so, it is important to understand how bioactive agents are transformed because this will determine their potential health benefits.

*Absorption* (*F*_A_): After travelling through the intestinal fluids and mucus layer, bioactive agents reach the surfaces of the epithelium cells where they may be absorbed by various passive or active transport mechanisms [78]. Some bioactives are expelled back into the intestinal fluids as a result of efflux transporters in the cell membranes. After absorption, bioactives are transported to the systemic system via the portal vein (hydrophilic bioactives) or lymphatic system (hydrophobic bioactives).

The overall bioavailability of ingested bioactives can therefore be controlled by designing delivery systems to increase *F*_B_, *F*_T_, and *F*_A_ [79]. In the following section, some of the major factors that may impact these parameters in plant-based milk substitutes are discussed.

### 4.3. Factors Impacting Overall Bioavailability

The composition and structure of emulsion-based delivery systems can be designed to control the major factors impacting the bioavailability of bioactive agents [80,81,82]:

*Transformation* (*F*_T_): The chemical stability of a bioactive within the GIT depends on its molecular environment. For instance, the chemical degradation of some bioactive agents occurs much more rapidly when they are dispersed in a hydrophilic environment than in a hydrophobic one [83]. Consequently, their degradation can be retarded by encapsulating them inside oil droplets [84]. Alternatively, natural antioxidants can be incorporated into a colloidal delivery system to inhibit the chemical degradation of any encapsulated bioactive agents that are prone to oxidation [85,86].

*Bioaccessibility* (*F*_B_): The bioaccessibility of oil-soluble bioactive agents depends on the formation of mixed micelles in the small intestine that are capable of solubilizing it [66,72]. The bioaccessibility of these substances can therefore be increased by ingesting them with a source of digestible lipids, such as triacylglycerols [87]. Small oil droplets are a particularly good source of digestible lipids because they are rapidly hydrolyzed within the small intestine [66]. A number of important factors impacting bioaccessibility are discussed in a later section.

*Absorption* (*F*_A_): Certain types of food-grade components promote the absorption of micronutrients by altering the intestinal cell membrane permeability or by interfering with active transport or efflux mechanisms, e.g., spices, medium chain fatty acids, and surfactants [88]. It may therefore be advantageous to incorporate these components into emulsion-based delivery systems to improve the absorption of encapsulated bioactive agents.

In summary, the composition and structure of the emulsion-based delivery systems used to fortify plant-based milk substitutes must be carefully designed to ensure good bioaccessibility, stability, and absorption of the encapsulated micronutrients [66,72].

### 4.4. Optimizing Delivery Systems for Fortifying Plant-Based Milk Substitutes

In this section, some of the major factors that would be expected to impact the bioavailability of the bioactive agents in fortified plant-based milk substitutes are highlighted. These factors have recently been reviewed in more detail elsewhere [66].

#### 4.4.1. Oil Phase Composition

**Figure 8:**
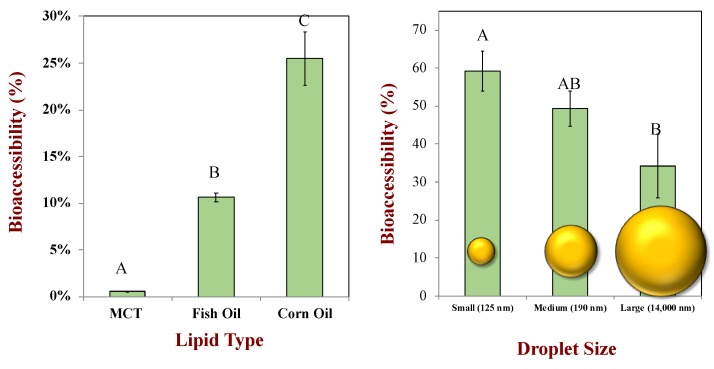
Impact of lipid type and oil droplet size on the extent of lipid digestion (%FFA) and β-carotene bioaccessibility from oil-in-water emulsions. Mean droplet diameters shown in nm. The letters above the bioaccessibility data show significant differences (*p* < 0.05). Adapted from Salvia–Trujillo et al. [92,97].

Numerous studies have shown that the composition of the oil phase in emulsion-based delivery systems can impact the bioavailability of encapsulated hydrophobic bioactives, such as oil-soluble vitamins and nutraceuticals [66]. The nature of the oil phase may impact bioavailability through a variety of mechanisms related to the protection, release, solubilization, and transport of the bioactives. First, if the bioactive agent is sensitive to chemical degradation, such as oxidation of unsaturated bioactives (like ω-3 fatty acids or carotenoids), then the oil phase may either promote or retard its degradation depending on its chemical nature. For instance, a highly saturated oil (like medium chain triglycerides, MCT) would be expected to limit degradation, while a highly polyunsaturated one (like fish oil) would be expected to promote it [86]. Moreover, any pro-oxidant impurities in an oil may promote the oxidation of a labile bioactive, thereby reducing its bioactivity [89,90]. Second, different oil phases may be digested at different rates and to different extents within the human GIT [91,92,93]. Typically, the carrier oil has to be digested before any encapsulated bioactives can be released. Consequently, the faster and more extensive the digestion, the higher the bioavailability. Third, the nature of the oil phase can impact the solubilization capacity of the mixed micelles generated within the small intestine [66]. The mixed micelles are formed from free fatty acids and monoacylglycerols coming from the digested fat, as well as bile salts and phospholipids from the body. The solubilization capacity of the mixed micelles for a particular bioactive depends on its dimensions relative to the dimensions of the hydrophobic domains inside the mixed micelles [94]. If the bioactive is too big to fit, then it will not be solubilized. Numerous studies have shown that the bioavailability of carotenoids (which are long hydrophobic molecules) is highly dependent on the oil type, being much higher for oils containing long chain fatty acids (corn oil) than those containing medium chain fatty acids (coconut oil or MCT) [95,96,97]. This is because the hydrophobic domains in mixed micelles formed from medium chain fatty acids are too small to accommodate the carotenoids. This effect is highlighted in Figure 8, which shows the impact of the oil phase type on the bioaccessibility of carotenoids in emulsion-based delivery systems. This phenomenon has consequences for the formulation of plant-based milk substitutes. For instance, if coconut milk was going to be fortified with large hydrophobic bioactive molecules (like carotenoids), it may be better to use a delivery system containing long chain fatty acids (such as sunflower or corn oil). It should also be noted that encapsulating bioactives in non-digestible oils (such as mineral, essential, or flavor oils) may also lead to a low bioavailability because few mixed micelles are generated [98,99]. As mentioned earlier, it has also been shown that oil-soluble vitamins (such as vitamin D) are more bioaccessible when incorporated into plant-based emulsions with an oil phase consisting of long-chain monounsaturated fats (such as corn oil) than long-chain polyunsaturated fats (such as flaxseed oil), which can be attributed to a similar region.

#### 4.4.2. Oil Phase Concentration

The bioavailability of hydrophobic bioactives also depends on the total amount of digestible oil inside a delivery system [97,100]. If there is too little oil present, then there are not enough mixed micelles generated to solubilize all of the hydrophobic bioactives present. Conversely, if there is too much oil present, then it may not be fully digested by the enzymes in the GIT, which means that all of the bioactives may not be released. Moreover, including too much oil in a food would increase the fat and calorie content, which may be undesirable from a nutritional point of view. Consequently, it is important to optimize the level of oil in a delivery system. For experience, we found that around 2%–6% oil is sufficient to ensure good bioaccessibility of highly hydrophobic bioactives (like carotenoids and oil-soluble vitamins).

#### 4.4.3. Droplet Size

The size of the oil droplets in a delivery system may also have a major impact on the bioavailability of encapsulated hydrophobic bioactives [66]. As the droplet diameter (*d*) decreases, the specific surface area (*A_S_*) of oil exposed to the surrounding aqueous phase increases: *A_S_* = 6*ϕ*/d (m^2^ per m^3^), where *ϕ* is the disperse phase volume fraction [19]. As a result, there is more surface area available for the digestive enzymes (such as lipase) to attach to, which increases the rate of lipid digestion. Consequently, the bioactives are released from the droplets more rapidly and solubilized in the mixed micelles more quickly. Moreover, all of the oil droplets tend to be digested by the end of the small intestine phase when they are small, which may not be the case when they are relatively large. A number of studies have shown that the bioaccessibility of hydrophobic bioactives increases as the oil droplet size decreases, e.g., carotenoids and oil-soluble vitamins [101,102,103,104,105]. This effect is highlighted in Figure 8, which shows the impact of oil droplet size on the bioaccessibility of carotenoids in emulsion-based delivery systems. As mentioned earlier, in a recent study, it was shown that the uptake of vitamin D was increased when the size of the lipid droplets in pea protein-stabilized nanoemulsions was decreased [67]. Other in vitro studies have also shown that the bioaccessibility of oil-soluble vitamins increases as the droplet size decreases [106]. Consequently, it may be important to ensure that any hydrophobic bioactives are encapsulated within small lipid droplets when they are incorporated into plant-based milk products.

#### 4.4.4. Emulsifier Type

The oil droplets in emulsion-based delivery systems may be coated by various kinds of natural or synthetic emulsifiers, such as proteins, polysaccharides, phospholipids, saponins, and small-molecule surfactants [20,21]. In vitro digestion studies have shown that the nature of the emulsifier-coating oil droplets can have a pronounced effect on the bioavailability of encapsulated hydrophobic bioactives [107,108,109,110]. This phenomenon may be due to a number of factors. If the emulsifier is highly surface active and present at a sufficiently high concentration, then it may be able to inhibit the adsorption of bile salts and lipase to the droplet surfaces, thereby reducing lipid digestion [111]. In practice, this is rarely possible because it requires high levels of synthetic surfactants. The most common way that emulsifier type alters lipid digestion is by altering the aggregation state of the oil droplets in the gastrointestinal tract [66]. Emulsifiers that lead to extensive droplet aggregation within the small intestine tend to inhibit lipid digestion, because there is a reduction in the surface area of the lipid phase exposed to the digestive enzymes when the droplets aggregate. Many protein-coated lipid droplets tend to aggregate in the stomach and small intestine, which can inhibit lipid digestion and bioactive release [109]. Conversely, many small molecule surfactants (such as Tween) tend to inhibit aggregation and lead to rapid digestion and bioactive release [109]. Consequently, it may also be important to carefully select the type of emulsifier used to stabilize the bioactive-fortified oil droplets in a plant-based milk.

#### 4.4.5. Food Matrix Effects

Finally, the composition and structure of the food matrix that surrounds the lipid droplets in an emulsion-based delivery system can have a pronounced effect on the bioavailability of encapsulated hydrophobic bioactives [112,113]. The nature of these food matrix effects is highly system dependent, but a number of food components are known to elicit these effects. For plant-based milk substitutes, the two most important components are calcium and dietary fiber. High levels of soluble calcium ions (Ca^2+^) have been shown to reduce the bioaccessibility of some hydrophobic bioactives because they promote precipitation of the anionic bioactive-loaded mixed micelles [114]. The presence of dietary fibers in a delivery system may increase or decrease the bioaccessibility of hydrophobic bioactives depending on its interactions with the lipid droplets and lipid digestion products. Dietary fibers that inhibit extensive aggregation of lipid droplets in the GIT can promote bioaccessibility by ensuring that the lipid phase is fully digested [115]. Conversely, dietary fibers that promote aggregation of the lipid droplets can reduce bioaccessibility by reducing the surface area of lipase to adsorb, and thereby retarding lipid digestion and bioactive release [116]. Dietary fibers may also inhibit lipid digestion and bioaccessibility by other mechanisms, including: Thickening or gelling the gastrointestinal fluids and therefore inhibiting mixing or mass transport processes; binding to critical gastrointestinal components (such as enzymes, bile salts, or calcium); and forming protective coatings around the lipid droplets that inhibit lipase adsorption [66,116].

## 5. Conclusions

An important trend in the modern food industry is the creation of more plant-based foods because of their perceived environmental, health, and ethical benefits. These products must be safe, affordable, convenient, and tasty if they are going to be adopted by a large fraction of the population. The main focus of this article was on the structural basis of the physicochemical, functional, sensory, and nutritional attributes of plant-based milk substitutes. These products are complex colloidal dispersions that contain various kinds of small particles dispersed within an aqueous medium, e.g., oil bodies, fat droplets, protein particles, or plant cell fragments. The nature of these colloidal particles, such as their composition, structure, size, interfacial properties, and interactions, ultimately determine the physical, functional, sensory, and nutritional attributes of plant-based milk substitutes, including their appearance, texture, mouthfeel, taste, stability, and nutrient bioavailability. Knowledge of the colloidal basis of plant-based milk substitutes can be used to enhance their quality attributes, functional performance, and healthiness.

## Figures and Tables

**Table 1:** Comparison of the physicochemical and structural properties of various plant-based milks: Apparent shear viscosity, flow index, mean particle diameter (D_32_ and D_43_), gravitational separation rate, and whiteness index. The data was extracted from a previously published work by Jeske and others [28].

Milk	Viscosity [mPa∙s]	Flow Index	D_3,2_	D_43_	Separation Rate (%h)	Whiteness Index
			[μm]	[μm]		
Almond	4.6	0.82	2.4	0.9	52.4	68.4
Almond	19.1	0.70	1.1	1.8	1.4	72.6
Almond	26.3	0.56	2.1	6.0	30.2	76.0
Almond	3.9	0.98	1.5	2.6	51.7	51.6
Cashew	5.6	0.97	2.3	29.2	27.5	65.6
Coconut	47.8	0.40	1.3	1.7	37.4	67.8
Hazelnut	24.8	0.67	1.5	2.2	1.3	56.3
Hemp	25.0	0.73	1.1	1.5	4.4	68.5
Macadamia	2.2	1.00	1.8	3.4	54.4	51.7
Oat	6.8	0.89	1.7	3.8	40.1	60.2
Quinoa	13.2	0.76	1.1	81.5	32.0	71.4
Rice	2.8	0.97	0.88	10.5	42.8	66.5
Brown Rice	2.2	1.00	0.63	0.72	50.9	63.5
Soy	7.6	0.90	0.94	1.3	11.3	70.3
Soy	3.5	1.00	0.80	1.0	8.6	74.5
Soy	2.6	1.00	0.85	1.0	13.3	69.3
Soy	6.0	0.92	0.94	1.2	22.6	74.6
Cow’s	3.2	1.00	0.36	0.60	3.9	81.9

**Table 2:** Some common strategies for improving the aggregation stability of plant-based milks based on knowledge of the nature of colloidal interactions.

Interaction Type	Strategy to Improve Stability
*Steric repulsion*	The steric repulsion between the particles can be increased by increasing the thickness of any adsorbed polymer layers.
*Electrostatic repulsion*	The electrostatic repulsion can be increased by increasing the magnitude of the charge or by reducing the ionic strength. This can often be achieved by controlling the pH and salt levels. For instance, the pH should be a long way from the isoelectric point.
*Hydrophobic attraction*	The hydrophobic attraction can be reduced by ensuring there are few non-polar patches on the surfaces of the colloidal particles. This might be achieved by controlling the degree of thermal denaturation of any proteins or by adding surfactants to cover non-polar patches.
*Bridging attraction*	The bridging attraction can be reduced by ensuring that there are no polymers or other substances that can simultaneously bind to the surfaces of multiple particles. Most importantly, charged polymers may link together oppositely charged particles.
*Depletion attraction*	The depletion attraction can be reduced by ensuring that the concentration of non-adsorbed polymers is below a critical level (which depends on their molecular weight and conformation).
